# Emerging Biomarkers in Vascular Cognitive Impairment and Dementia: From Pathophysiological Pathways to Clinical Application

**DOI:** 10.3390/ijms20112812

**Published:** 2019-06-08

**Authors:** Virginia Cipollini, Fernanda Troili, Franco Giubilei

**Affiliations:** S. Andrea Hospital, NESMOS Department, Faculty of Medicine and Psychology, Sapienza University of Rome, Via di Grottarossa 1035, 00189 Roma, Italy; f.troili86@gmail.com (F.T.); franco.giubilei@uniroma1.it (F.G.)

**Keywords:** vascular disease, vascular dementia, vascular cognitive impairment, peripheral biomarkers, blood biomarkers, CSF biomarkers

## Abstract

Vascular pathology is the second most common neuropathology of dementia after Alzheimer’s disease (AD), with small vessels disease (SVD) being considered the major cause of vascular cognitive impairment and dementia (VCID). This review aims to evaluate pathophysiological pathways underlying a diagnosis of VCID. Firstly, we will discuss the role of endothelial dysfunction, blood-brain barrier disruption and neuroinflammation in its pathogenesis. Then, we will analyse different biomarkers including the ones of inflammatory responses to central nervous system tissue injuries, of coagulation and thrombosis and of circulating microRNA. Evidences on peripheral biomarkers for VCID are still poor and large-scale, prospectively designed studies are needed to translate these findings into clinical practice, in order to set different combinations of biomarkers to use for differential diagnosis among types of dementia.

## 1. Background

### Vascular Cognitive Impairment and Dementia: An Umbrella Term

In recent years, many efforts have been made to achieve consensus in defining cognitive impairment with vascular pathology [1] and the term vascular cognitive impairment and dementia (VCID) is recently being used as an umbrella term [2,3]. Despite the variable prevalence estimated in the literature, varying according to the employed diagnostic criteria, VCID is recognized as the second most common cause of dementia after Alzheimer’s disease (AD) [4]. The term VCID refers to a group of different diseases characterized by cognitive impairment caused primarily by cerebrovascular disease (CVD). This includes the full spectrum ranging from vascular mild cognitive impairment (MCI) to Vascular Dementia (VaD) [4]. Recently, due to this heterogeneity, the Society for Vascular Behavioral and Cognitive Disorders (VASCOG) produced criteria for vascular cognitive disorder [5] which were harmonized with the DSM-5 criteria [6]. Core features include: stepwise progression, focal neurological signs and symptoms, unequal distribution of cognitive deficits, history of multiple ischemic strokes, neuroimaging evidence of cerebrovascular disease and temporal relationship between CVD and cognitive impairment [7].

Nowadays, there is a strong scientific rationale for linking CVD to cognitive impairment, including the link between age-related cerebrovascular changes and dysregulation of cerebral perfusion, blood–brain barrier (BBB) function, and neurovascular unit (NVU) coupling. The range of neuropathological mechanisms underlying a diagnosis of VCID include: post-stroke impairment, small and large vessel diseases, and cases of mixed-pathology, with CVD that interacts with AD pathology [8]. 

Initially, vascular dementia was thought to be related to the volume of multiple strokes, condition called multi-infarct dementia (MID) [9]. Then, the use of brain imaging revealed a high incidence of white matter damage not necessarily related to strokes. In this regard, there was a growing awareness of the importance of slowly progressive changes in the brain related to small vessels disease (SVD) [10]. It is now thought by many investigators that SVD is the major cause of VCID and the most common pathology found in the elderly, doubling the chances that neurodegenerative pathology would lead to dementia [11]. 

In the past years, research on biomarkers has mainly focused to relate candidate markers to specific neuroimaging features, such as lacunar infarcts and white matter hyperintensities (WMHs) in population-based studies or in patients with a high risk of vascular factors. Other studies have compared possible biomarkers according to the different subtypes of VCID disorders. Indeed, advances in neuroimaging and biochemical analysis of blood and cerebrospinal fluid (CSF) provide unique multimodal biomarkers to stratify VCID patients [12,13].

In this review, we will briefly summarize the evidences on emerging blood and CSF biomarkers related to VCID, particularly with SVD, a common, fairly homogeneous, but often under-recognized histopathologic subtype of VCID. Firstly, we will consider the pathophysiological pathways underlying a diagnosis of VCID and we will discuss the role of endothelial dysfunction, blood-brain barrier disruption and neuroinflammation in its pathogenesis. Then we will analyse different biomarkers including biomarkers of inflammatory responses, biomarkers of central nervous system tissue injuries, biomarkers of coagulation and thrombosis, and circulating microRNA (miRNA).

## 2. Methods

For this narrative review, we conducted a comprehensive search on Medline and PubMed databases for studies published until 31st March 2019. The keywords used in the current search were: “vascular dementia” or “vascular cognitive impairment” or “subcortical small vessel disease” AND “biomarkers”, “inflammation”, “blood brain barrier”, “endothelial dysfunction”, “microRNA”. We used filters for English language and selected manuscripts not older than 10 years. Two of the authors, VC and FT, separately selected relevant abstracts and critically reviewed them. The full text of 197 relevant papers were taken into account. Moreover, 24 reviews from the last 5 years that could be of interest for our work were considered. The proposal on current status of biochemical markers in VCID was reviewed by the group.

For the tables compilation filters for publication dates not older than the last 5 years, only for human subjects and only in English language were applied, obtaining 326 articles to analyse. We then excluded all the reviews and not pertinent topics by reading article abstracts, and then obtained all the salient full texts (49 articles in total) (Table 1 and Table 2).

## 3. Pathophysiological Pathways of VCID

The heterogeneity of CVD makes it challenging to elucidate the neuropathological substrates and mechanisms of VCID. VCID is an entity whose heterogeneous clinical manifestations are due to a substrate of multiple pathogenic and structural factors. Nevertheless, histopathologic evidence, obtained by biopsy or autopsy, is essential in each guideline to make a diagnosis of definite VCID [14]. Decreased Cerebral Blood Flow (CBF) is the major cerebral hemodynamic alteration in VCID and pathologies, which cause reduction in global CBF, such as atherosclerosis and arterial stenosis are involved [15]. Factors that define the subtypes of VCID include the nature and extent of vascular pathologies (such as ischemic infarcts, hemorrhages and white matter changes), the degree of involvement of extra and intracranial vessels and the anatomical location of tissue changes. Typical neuropathological changes of AD such as amyloid plaques and neurofibrillary tangles may also be found at the pathological examination of VCID and may contribute to cognitive dysfunction [5,16].

Neuroimaging and neuropathology studies have established that clinically silent CVD is sufficient to cause relevant cognitive impairment in the absence of stroke [5]. SVD, characterised by arteriolosclerosis and lacunar infarcts, causes cortical and subcortical microinfarcts, which appear to be the most robust pathological substrates of VCID [14]. Indeed, diffuse damage to the white matter (ischemic leukoencephalopathy) is the most common pathology in SVD [66]. It is usually most severe in frontal and occipital regions and the area involved shows loss of both myelin and axons as well as chronic inflammatory infiltrates [67]. The mechanism underlying white matter lesions (WMLs), which lead to demyelination and gliosis, involves a multifactorial process, including BBB disruption, hypoxia and hypoperfusion, oxidative stress, neuroinflammation and alteration on NVU coupling [15]. In addition to lacunar infarcts and WMLs, brain atrophy and visible Enlarged Perivascular spaces (EPVS) are other common radiologic features of SVD. Their relationship to vascular cognitive impairment, however, is not well established [68]. Recent pathological data show that SVD may be linked to the dysfunction of the glymphatic pathway that might be involved in the clearance of mis-aggregated proteins. On this basis, EPVS, identifiable on brain MRI, have been described as a potential biomarker of neurovascular dysfunction and of impaired clearance of proteins [68,69]. Finally, hemorrhagic manifestations such as cerebral microbleeds or superficial siderosis are other possible causes of cognitive impairment in SVD [14].

As we mentioned above, SVD is one of the major causes of VCID and it is usually a sporadic disease caused by age and hypertension [70], however there are also monogenic forms of SVD. The most common form is the Cerebral Autosomal Dominant Arteriopathy with Subcortical Infarcts and Leukoencephalopathy (CADASIL), caused by mutations in the NOTCH3 gene [71]. Other rare forms include the Cerebral Autosomal Recessive Arteriopathy with Subcortical Infarcts and Leukoencephalopathy (CARASIL), an arteriopathy caused by mutations in the HTRA1 gene [72] and the Cathepsin A–Related Arteriopathy with Strokes and Leukoencephalopathy (CARASAL), an autosomal dominant disease caused by heterozygous mutation in the CTSA gene encoding cathepsin A [73]. Moreover, other adult-onset genetic leukoencephalopathies have recently been described, such as Hereditary Diffuse. Leukoencephalopathy with Spheroids (HDLS), caused by mutations in the CSF1R (Colony Stimulating Factor-1 Receptor) gene and responsible of approximately 10% of cases of adult-onset leukodystrophy [74]. It is important to recognize these forms of SVD to make an accurate diagnosis and a correct family counseling.

## 4. Endothelial Dysfunction 

The endothelium is a monolayer of endothelial cells that separates the tissues from the circulating blood. It is involved in several vascular processes and functions. The term endothelial dysfunction indicates a condition in which the endothelium is damaged and can develop proinflammatory, provasoconstriction and procoagulation states [75]. 

As we mentioned above, most of VCID is caused by cerebral hypoperfusion [76]. Cerebrovascular endothelial cells are usually the foremost in bearing the attack of hypoperfusion. The subsequent endothelial dysfunction could lead to many processes such as an increase in BBB permeability, an exposure of neural cells to harmful substances, a rise of inflammatory environment and a dysfunction of neurovascular coupling. The result is an activation of glial cells in the CNS, which lead to WMLs and neuron damage [77]. An Australian study showed that reduced endothelial integrity was associated with increasing WMHs severity [78]. Moreover, a significant decrease in endothelial function and in BBB integrity are present in areas of WMHs compared with normal white matter [78]. This hypothesis is supported by neuropathological studies, showing that WMLs are characterized by the expression of hypoxia-related molecules and endothelial markers [79]. Cerebral hypoperfusion up-regulates the expression of adhesion molecules such as intercellular adhesion molecule-1 (ICAM-1) and vascular cell adhesion molecule-1 (VCAM-1), markers of endothelial cell activation [80]. Finally, endothelial dysfunction promotes oxidative stress from unbalanced free radical formation, which leads to peroxynitrite formation, lipid peroxidation, protein modification, matrix metalloproteinases (MMPs) activation, and DNA damage [13]. 

## 5. Blood-Brain Barrier and Choroid Lexus Breakdown

The BBB isolates the central nervous system (CNS) from the systemic circulation and it is essential in maintaining the optimal microenvironment in the CNS. It is maintained by the interplay between endothelial cells, pericytes, and astrocytes [81]. 

The choroid plexus is an epithelial monolayer that forms the blood-cerebrospinal fluid barrier. The main function of choroid plexus is to produce CSF, but it also acts as a neuro-immune interface by integrating signals from the brain with signals coming from the circulation. The CSF/serum albumin ratio is the gold-standard measure of BBB and choroid plexus integrity, with increases in this ratio indicating increased permeability. Disruption of BBB and choroid plexus functions, followed by a rise of neuroinflammatory molecules coming from blood circulation, may promote brain damage [67]. These events may be involved in disease progression in both VCID and AD [82].

Nowadays, it is well known that BBB permeability is altered in patients with cerebral microvascular disease. A recent meta-analysis [83] revealed that BBB permeability was increased further in patients with either VaD or AD compared with age-matched healthy controls. Moreover, the permeability was higher in VaD compared with AD.

## 6. The Role of Inflammation

**Figure 1:**
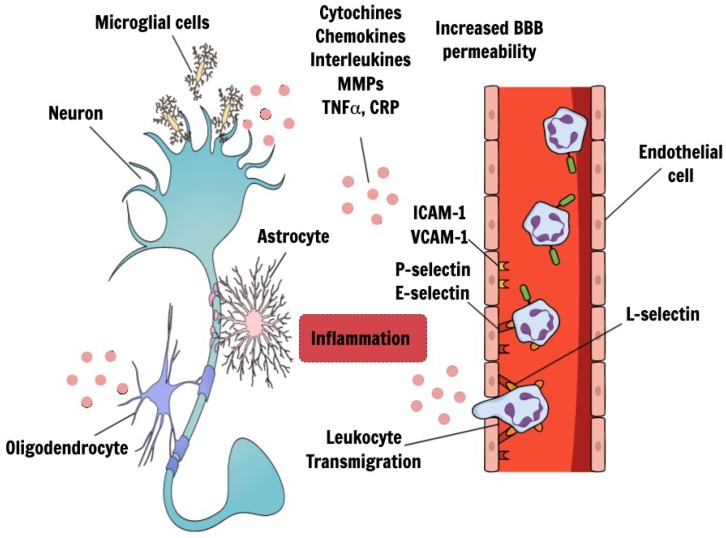
Endothelial dysfunction and cerebral inflammatory response in VCID. Endothelial dysfunction, observed in VCID, could lead to an increase in BBB permeability, a subsequent exposure of neural cells to harmful substances and a rise of inflammatory environment. The result is an activation of glial cells in the CNS, which finally lead to neuron damage. During inflammatory response, leukocytes are recruited from the circulation into the extravascular space. The activation of endothelial cells lead to the expression of adhesion molecules on their surfaces, allowing the binding to reciprocal molecules on the surfaces of circulating leukocytes. Indeed, activated endothelial cells up-regulate the expression of adhesion molecules such as intercellular adhesion molecule-1 (ICAM-1), vascular cell adhesion molecule-1 (VCAM-1) and selectins (P-selectin, E-selectin). L-selectin is mainly expressed on leukocytes. The increased BBB permeability lead to infiltration of inflammatory factors like interleukins, chemokines, cytochines, MMPs, TNFα, TLR4, CRP. Upon entry into the brain, these inflammatory factors can exacerbate white matter damage (demyelination, axonal loss, oligodendrocyte degeneration), cause neurodegeneration and cell death as well as enhance neuroglial inflammation. (VCID: vascular cognitive impairment and dementia; BBB: blood–brain barrier; CNS: central nervous system; MMPs: TNFα: Tumor necrosis factor; CRP: C-reactive protein).

Inflammation has been recognized to have a crucial role in the pathogenic mechanisms of cerebrovascular and neurodegenerative diseases [84]. It is not yet known whether inflammation is a primary driver of VCID and whether this neuroinflammation is triggered by intrinsic or systemic processes [80]. Certainly, clinical observations suggest that an interplay between CNS and peripheral inflammation exists [85]. Microvascular inflammation, as outlined previously, is a common feature of hypoperfusion models with markers of chronic inflammation and endothelial activation, leading to increased BBB permeability and to infiltration of inflammatory factors like interleukins (ILs), MMPs, Tumor necrosis factor (TNFα), toll-like receptor 4 (TLR4), C-reactive protein (CRP). Upon entry into the brain, these inflammatory factors can exacerbate white matter damage (demyelination, axonal loss, oligodendrocyte degeneration), cause neurodegeneration and cell death as well as enhance neuroglial inflammation [86] (Figure 1). Indeed, clinical and experimental studies have shown that white matter damage is not merely a consequence of chronic oxygen deprivation but is induced and sustained by pro-inflammatory environments [85]. In the hippocampus, as a result of the inflammatory cascade, it was observed an impaired neurogenesis, a dysregulation of progenitor cell proliferation and an alteration of synaptic plasticity and dendritic spine density [86].

Several other processes are promoted by inflammation and might contribute to the pathogenesis of VCID such as atherogenesis and platelet aggregation. Cytokines, leukocyte adhesion molecules, chemokines, growth factors, and lipids mediate these processes [13]. 

## 7. Biomarkers

Identifying a diagnostic biomarker in VCID would be of paramount interest in order to contribute to a better understanding of the mechanisms underlying this affection. The goal of diagnostic biomarkers development in VCID is to identify patients at early stage when treatment may be effective in blocking the progressive damage to the white matter. Nevertheless, it may be also desirable to find biomarkers that allow the clinician to follow the natural history of the disease, in terms of severity and prognosis. 

An accurate biomarker—such as a protein, nucleic acid, or metabolite—should represent a quantification of a definite biological state. Due to its heterogeneity, VCID may not have a uniform presentation and a linear progress over time, so biomarkers may differ depending on aetiology, clinical phase and histopathologic involvement. Given these premises, it is likely that a single biomarker might not be adequate to identify underlying complexities that are at the basis of cellular changes linked to VCID. Furthermore, it has to be reminded that in clinical practice a circulating biomarker has to be easily assessed with low cost, and patient-friendly procedure. In this light, several families of candidate biomarkers have been studied in relation to VCID and multiple approaches have been developed to identify biomarkers [87]. To facilitate a thorough comprehension of their significance in terms of aetiology, they could be categorized in four subgroups:

(1) Biomarkers of Inflammatory Response;
(2) Biomarkers of Central Nervous System Tissue Injuries;
(3) Biomarkers of Coagulation and Thrombosis;
(4) Circulatory miRNAs.

### 7.1. Biomarkers of Inflammatory Response

Strong evidences suggest that chronic inflammation is involved in the pathogenesis of several conditions such as stroke, dementia, cardiovascular disease and atherosclerosis [88]. However, the data regarding the association between inflammatory biomarkers and VCID are still inconclusive. 

Considering SVD, several studies found an association between inflammatory biomarkers and the progression of the disease. In this context, the vascular damage observed in the brain may be promoted and sustained by cytokines, acute-phase proteins, endothelial cell adhesive molecules, and other immune-related proteins (Figure 1). However, due to the lack of consensus on analytic methods, the results are often inconsistent [89]. Traditionally, the most commonly studied biomarkers in this field are interleukin-6 (IL-6) and CRP [90].

IL-6 is secreted as a pro-inflammatory cytokine by tunica muscularis cells of the blood vessels. In MRI studies, a positive correlation between IL-6 and WMHs has been detected [91]. As a response to IL-6, the liver synthesizes the CRP, which increases BBB permeability. On these bases, it has been suggested that CRP may mirror the inflammatory process in the brain and described that higher CRP levels are associated with increased risk of dementia [92]. It has also been reported an association between typical MRI findings in VCID such as WMHs, ischemic lacunes and EPVS and higher CRP levels [92,93,94]. Furthermore, a rapid decline in CRP levels predicts a healthier white matter microstructure [95]. However, the association between CRP and WMHs was reconsidered by the adjustment of cardiovascular factors [96]. In contrast to these results, other studies did not find any associations between CRP or other cytokines and the severity of WMHs volume in VCID [89].

Inflammatory biomarkers other than CRP or IL-6 may be relevant for WMHs in VCID. Indeed, higher levels of several endothelial dysfunction markers such as soluble E-selectin, soluble intercellular adhesion molecule 1, soluble P-selectin, and soluble vascular cell adhesion molecule-1 have been related with WMHs in cross-sectional studies [97,98]. Moreover, Gu et al. [89] found that alpha 1-antichymotrypsin (ACT), which plays a role in cell adhesion and endothelial dysfunction, was associated with both WMHs severity and WMHs progression [89]. 

MMPs are other biomarkers of inflammation. They are a family of 26 extracellular matrix-degrading enzymes that attack the basal lamina and tight junctions of the cerebral endothelial cells, causing BBB dysfunction. They have been identified in pathological tissues from animal models of VCID and in human tissues [59,99]. Some MMPs, such as MMP-2, are normally present in the CSF. Others (mainly MMP-3 and MMP-9) are present at very low levels in the CSF in the absence of an inflammatory state [100]. Some studies have demonstrated that the evaluation of MMPs in CSF have high validity in discriminating VCID from cognitive impairment of primarily neurodegenerative etiology [12,101,102]. Indeed, elevated CSF levels of MMP-9 are present in patients with VCID and mixed AD/VCID but not in AD [12]. MMPs were explored also in plasma levels. A work by Duits et al. [19] showed how they can help differentiate VaD from AD. Given these evidences, MMPs could be promising biomarkers in the classification of the different types of dementia. 

### 7.2. Biomarkers of Central Nervous System Tissue Injuries

Numerous biomarkers have been associated with CNS tissue injury, including neuron specific enolase (NSE), *N*-methyl-d-aspartate receptor (NMDAR) antibodies, protein S-100B, and myelin basic protein (MBP). 

NMDAR are both ligand-gated and voltage dependent and are involved in long-term potentiation in learning processes of the brain [103]. Autoantibodies directed to some NMDAR subtypes have been mainly studied in acute cerebrovascular events, but also in post-stroke cognitive impairment. Busse and colleagues [53] examined the prevalence of NR1a NMDAR autoantibodies in both serum and CSF of subjects affected by AD, SVD and healthy subjects. The results of the study showed the presence of NMDAR IgM, IgG, and/or IgA autoantibody titers in serum of patients with SVD. However, the results were not conclusive, because the seroprevalence of NMDAR-directed autoantibodies was age related [53].

MBP is a hydrophilic protein found in myelin sheaths, whose serum levels have been found in several neurological diseases. At this regard myelin loss has been found in different types of dementia. Literature data suggest that myelin loss, secondary to hypoxic–ischemic damage, may evolve in parallel with shrunken oligodendrocytes in VaD [104,105].

S110B is an astroglial protein that has been studied as a serum marker for cerebral injury and disruption of the BBB. It has been studied as an independent predictor and diagnostic marker for stroke, VCID, and AD. However, no significant difference in S100B CSF levels was found between AD and VCID patients [106].

Neurofilament light chain (NfL) is an emerging blood and CSF biomarker for neuroaxonal damage. Its role has been explored in multiple neurological diseases affecting the elderly population, such as motor neuron disease, AD and frontotemporal dementia [56]. A recent study found, in both sporadic SVD and CADASIL patients, an association between serum NfL levels and impaired processing speed performance. Furthermore, CADASIL patients showed a strong and independent association between serum NfL levels and measures of focal neurological deficits and disability [107].

The results of another study suggest that in CADASIL the serum NfL levels correlate with disease severity, disease progression and 17-year survival [108]. Given these evidences, in CADASIL patients serum NfL could be a promising biomarker to monitor disease course and a possible target for new therapies.

Finally, evidences on CSF levels of Aβ and Tau proteins reveal how they may be of help in differentiating VCID from AD, even if there is a certain degree of overlap between AD and non-AD dementias, possibly reflecting underlying mixed pathologies [54,57,59,60,61].

### 7.3. Biomarkers of Coagulation and Thrombosis

It is well known that coagulation and fibrinolytic pathways are involved in cerebrovascular diseases. However, little is known about the role of haemostatic biomarkers in VCID with most of the studies yielding discordant evidences [13].

Biomarkers identifying the coagulation cascade have been linked to stroke, VaD and AD: such biomarkers include, among the others, fibrinogen and lipoprotein-associated phospholipase A2 (Lp PLA2).

Fibrinogen is a soluble plasma glycoprotein involved in the coagulation cascade. Some studies suggest that high fibrinogen levels correlate with increased risk of dementia, both AD and VaD [109,110]. This is supported by a study on MCI patients, which observed that hyperfibrinogenemia was associated with an increased risk for developing VaD [111].

Lp PLA2 is an enzyme expressed primarily by leukocytes that influence the degradation of platelet-activating factors to inactive factors. It plays a crucial rule in the metabolism of low-density lipoprotein to proinflammatory proteins. Lp PLA2 is expressed in the necrotic core of atherosclerotic plaques, thus resulting a valid biomarker of atherosclerosis and plaque inflammation. However, an increased risk of dementia has also been associated with eleveted Lp PLA2 activity [112]. 

It is known that prothrombotic status is associated with cardiovascular risk. Moreover, tissue factor and thrombomodulin, markers of endothelial activation and damage, have been associated with the extent of leukoaraiosis in SVD [113].

Several studies found an association of high plasma levels of von Willebrand factor, a glycoprotein expressed by endothelial cells after tissue damage, with the number of lacunes, periventricular WMHs, and WMHs burden [114,115]. Moreover, in CADASIL patients, von Willebrand factor was significantly higher than in controls [116].

Different authors described an association between high homocysteine levels, depending on metabolism of dietary methionine, and WMHs or silent lacunar infarcts [75]. In a large cohort of CADASIL patient, hyperhomocysteinemia was associated with an increased risk of migraine [117]. 

Endothelial progenitor cells (EPCs) and circulating progenitor cells (CPCs) are bone marrow-derived cells involved in the maintenance of the structure and homeostasis of the endothelium [118]. Lower levels of EPCs are a strong predictor of cardiovascular events and some studies reported lower levels of EPCs in CADASIL and sporadic SVD patients [118]. These results were confirmed in a larger cohort of CADASIL patients [116]. About the CPCs levels in CADASIL patients, they were significantly correlated with a more severe decrease of both cognitive and motor performances [118] as well as with neuroimaging findings [116]. 

### 7.4. Circulating microRNA

miRNAs are small, noncoding, single-stranded RNA molecules that negatively regulate gene expression via translational inhibition or mRNA degradation. Their biological importance within organisms at all levels of the evolutionary scale has been demonstrated [119]. miRNAs genes are excised from exons and introns or other intergenic regions of the genome [120]. 

An alteration of miRNAs expression has been observed in several human diseases and it may play a role in CVD, improving the gene-regulatory processes [121]. For these reasons, research on peripheral biomarkers in various human diseases is considering miRNAs [122]. Circulating miRNAs have been detected in plasma, serum, whole blood, urine, saliva, sweat, breath, and CSF, and, because of their small molecular size, they cross biological barriers (e.g., blood/brain, blood/placenta) [123]. Therefore, they have the potential to serve as disease biomarkers, being reported to be reproducible and stable among people [124]. Increasing number of miRNAs have been proven to be critical for the pathogenesis of neurological diseases [125]. Many studies have shown that, after CNS injury, altered miRNAs may activate processes that stimulate neuronal death through inflammation, apoptosis, and oxidative stress [126].

In a recent work by Ragusa et al. [127], miRNA such as miR-10b*, miR29a-3p and miR-130b-3p were downregulated in both VaD and AD patients, but the levels of miR-130b-3p were lower in AD than in VaD patients. Furthermore Dong et al. [18] found that a panel of other three miRNA (miR-31, miR-93 and miR-146a) significantly upregulated in VaD patients, could be used to discriminate AD from VaD.

With the purpose of differentiating VaD from AD, a small exploratory investigation by Sørensen et al. [128] focused on the analysis of miRNA in CSF of patients affected by AD and by other types of dementia. In this exploratory study, deregulated miRNAs in seem to be associated with target genes related to AD pathology, suggesting that miRNAs are interesting candidates for AD biomarkers in the future. 

Finally, Prabhakar et al. [39] revealed that four miRNAs (miR-409-3p, miR-502-3p, miR-486- 5p and miR-451a) could be used as valuable biomarkers to differentiate SVD patients from healthy controls and might serve as diagnostic biomarkers for this disease. Currently, information regarding miRNAs in VCID studies is limited, but the potential diagnostic value of miRNAs is very promising. 

## 8. Discussion

We have reviewed blood and CSF biomarkers that have been related to VCID, with both sporadic and genetic SVD. A summary of the best emerging biomarkers is presented on Table 1 and Table 2. Evidences from the literature are heterogeneous and mostly inconclusive. This heterogeneity is mainly explained by the differences of studies design, including different cohorts of patients analyzed and different sample sizes. However, it has to be remarked that VCID is a complex syndrome, possibly resultant from a multifactorial source of pathogenic processes. Therefore, a panel of biomarkers that reflects the different pathophysiological characteristics of the disease, might be needed to understand the contributions of each pathway involved in its pathogenesis.

From the data collected, the stronger results regard inflammatory biomarkers and molecules involved in endothelial dysfunction and coagulation cascade, although some of them have also been described to be altered in AD. This is the case of serum NfL, that is considered a potential circulating biomarker for both sporadic SVD and CADASIL burden [107]. It is a non-specific biomarker for neuroaxonal damage and higher serum levels have been reported also in AD patients. Commonly, the overlap of these biomarkers in VCID and AD may reflect the presence of concomitant AD and VCID neuropathological alterations.

Regarding endothelial circulating biomarkers including EPCs and CPCs, a possible limitation is that their levels in blood samples depend on the status of systemic endothelium and not only of cerebral endothelium [75].

Currently, data regarding miRNAs in VaD and Post Stroke Dementia (PSD) studies are limited, but the potential diagnostic value of miRNAs is very promising. In spite of the rapidly growing number of publications on diagnostic applications of circulating miRNAs, their use for screening of CNS diseases is in early stages of development. One factor impeding the progress in this field is the difficulty of comparing the data reported by different groups due to the use of different search methods and different techniques for miRNA measurement as well as the lack of data normalization. 

Another critical issue of the use of circulating miRNAs as biomarkers for diagnosis and e progression of a disease is that the pathologic processes that support the onset of the disease may change during disease progression. Furthermore, some processes, such as destruction of synapses, are common for different pathologies, including normal brain aging and neurodegenerative diseases, making the concept of differential diagnosis in CNS disease more complicated. The detection of specific miRNAs for brain regions and disease stage, preferably with longitudinal studies, might be useful in following the natural history of the disease and might facilitate the development of screening, predictive.

In this review, we do not include the analysis of biomarkers related to different clinical symptoms or VCID phenotypes. We only reported the results coming from studies on monogenic form of SVD, where the population involved is more homogeneous. Of course, biomarkers alone are often not enough to draw conclusions on a diagnosis, so they use should be considered as a support tool for a more thorough patient assessment, in a “difficult” diagnosis.

## 9. Conclusions and Future Perspectives

VCID is a common neurocognitive disorder still under-recognized in clinical practice and not well studied in research contexts. Evidences about CSF and blood-circulating proteins highlight the involvement of several interrelated mechanisms in the pathogenesis of VCID, including neuroinflammation, haemostasis, lipid metabolism and endothelial dysfunction. Starting from these mechanisms, emerging biomarkers have been studied. Most of analyzed studies show significant cross-sectional associations with neuroimaging or cognitive outcomes, but their utility in clinical setting remains to be established.

An important factor that restrict the applications of these biomarkers is the difficulty of comparing the data reported by different groups, due to the use of different laboratory techniques and methods of measurement. A codification for standardized procedures and methods would be of great value to further investigations.

In conclusion, further studies are needed to better understand the underlying mechanisms of tissue injury in VCID. Moreover, there is the necessity to search for an optimal panel of biomarkers with high sensitivity and specificity through a collaborative international network with harmonized protocols and procedures.

## Figures and Tables

**Table 1:** Blood biomarkers and VCID.

Author, Year [Reference]	Population	Biomakers	Outcome	Results
Murr J et al., 2014 [17]	670 cognitively normal subjects	Plasma oxidized low-density lipoprotein (OxLDL) levels	Risk Factor follow-up: 4.8 years (2.2–10.3)	No association between OxLDL and risk of all-cause dementia, AD, and vascular dementia or subtypes was found.
Dong H et al., 2015 [18]	127 AD patients, 30 MCI and 30 VaD patients.	Serum circulating miRNAs	Differential diagnosis	The panel of miR-31, miR-93 and miR-146a can be used to discriminate AD from VaD.
Duits FH et al., 2015 [19]	52 AD patients, 24 VaD patients, 26 cognitively normal subjects.	Plasma and CSF levels of MMP2, MMP9, MMP10, TIMP1 and TIMP2. CSF levels of amyloid-β(1–42) (Aβ42), tau, and tau phosphorylated at threonine-181 (p-tau).	Differential diagnosis	AD patients showed higher plasma MMP2 levels compared to VaD patients (*p* < 0.05).
Hatanaka H et al., 2015 [20]	72 AD, 27 VaD, 24 Mixed Dementia (MD) patients and 53 cognitively normal subjects.	Plasma levels of diacron reactive oxygen metabolite (dROM) and biological anti-oxidant potential (BAP).	Differential diagnosis	The dROM levels were significantly higher in the AD and MD groups than in the control group. The BAP levels were significantly lower in the MD group than in the control, AD and VaD groups.
Hilal S et al., 2015 [21]	41 cognitive impairment with no dementia (CIND) subjects and 46 demented subjects with burden of cerebrovascular diseases (CeVD); 37 CIND and 34 cases with dementia without CeVD; 35 cognitively normal subjects.	Plasma NTpro-BNP and high sensitivity cardiac troponin T (hs-cTnT) levels.	Differential diagnosis	Plasma concentrations of hs-cTnT were associated significantly with CeVD in both CIND and dementia. NTpro-BNP was associated with dementia with CeVD
Teunissen CE et al., 2015 [22]	295 subjects including healthy controls (*n* = 65), patients with subjective memory complaints (*n* = 99), patients with AD (*n* = 100), and patients with vascular dementia (*n* = 31).	Serum leptin levels	Differential diagnosis	Serum leptin levels are not altered in AD or vascular dementia patients compared to healthy controls and were not related to cognitive decline.
Wang C et al., 2015 [23]	10 cognitively healthy subjects and 10 age-matched VaD subjects.	Differentially expressed proteins (DEPs)	Differential diagnosis	High-degree proteins were detected in the protein-protein (PPI) interaction network, such as ATP5B (ATP synthase subunit β) in VaD.
Castellazzi M et al., 2016 [24]	232 MCI, 65 VaD, 175 AD, 88 MD, 104 Multiple Sclerosis (MS) patients, 165 cognitively healthy controls.	Serum High-density lipoprotein (HDL)-bound paraoxonase-1 (PON-1) levels	Differential diagnosis	Serum arylesterase, but not paraoxonase, levels were significantly lower in patients affected by MCI, VaD, AD, MD as well as those with MS as compared to healthy controls.
Chen Z et al., 2016 [25]	52 subcortical ischemic vascular disease patients with no dementia (SIVDND), 55 patients with mild cognitive impairment (SVMCI), 54 patients with vascular dementia (VaD), 54 cognitively healthy controls.	Serum thyroid-stimulating hormone (TSH), total triiodothyronine (TT3), free triiodothyronine (FT3), total thyroxine (TT4) and free thyroxine (FT4), thyroglobulin antibody (TGA), and antithyroid peroxidase antibody (TPO-Abs).	Correlation with cognitive status	Serum TT3 and FT3 levels decreased, whereas serum TSH level increased, with the decline in cognitive functions in SIVD.
Dukic L et al., 2016 [26]	235 participants, divided in 4 groups: AD (*N* = 70), VaD (*N* = 67), MCI (*N* = 48) and Cognitively Healthy Participants (*N* = 50).	Serum kallikrein 6 (KLK6), clusterin (CLU), adiponectin (ADPN) and interleukin-6 (IL-6)	Differential diagnosis	Serum concentrations of KLK6, CLU and ADPN did not differ between AD, VaD, MCI and cognitively healthy control group of participants, whereas IL-6 was significantly higher in VaD patients than in AD, MCI and healthy individuals.
Horvath I et al., 2016 [27]	40 nondemented controls, 11 stable mild cognitive impairment (SMCI), 6 MCI due to AD (MCI-AD), 40 AD and 7 VaD.patients.	CSF concentration of Aβ1−42, S100A8, S100A9 and Tau.	Differential diagnosis	The S100A9 and Aβ1−42 levels correlated with each other: their CSF content decreased already at the SMCI stage and declined further under MCIAD, AD, and VaD conditions.
Kitagawa K et al., 2016 [28]	466 cognitively healthy subjects	serum high-molecular-weight (HMW) adiponectin level	Risk factor (median follow-up period: 6.9 years)	Risks of dementia in patients with high versus low HMW adiponectin levels were almost identical. No association was found between adiponectin levels and Alzheimer’s disease dementia or vascular dementia in the whole group or amongst men and women separately.
Levada OA et al., 2016 [29]	21 patients with AD; 22 patients with subcortical vascular dementia; 16 cognitively healthy subjects.	Plasma levels of brain-derived neurotrophic factor (BDNF)	Differential diagnosis	At baseline there was lower BDNF levels in both AD and VaD groups, which was significant only in subjects with AD.
Mirza SS et al., 2016 [30]	357 AD patients; 32 VaD patients.	Serum N-terminal pro B-type natriuretic peptide (NT-proBNP) levels	Risk factor	Higher NT-proBNP was associated with a higher risk of dementia, with a particularly strong association with vascular dementia.
Nilsson ED et al., 2016 [31]	374 incident dementia cases: 120 AD patients, 84 VaD and 102 MD patients.	Plasma level of copeptin	Risk factor (median follow-up period: 4.2 years)	Baseline level of copeptin predicted incident VaD.
Pan X et al., 2016 [32]	Exploration phase: 338 control subjects and 43 AD patients; Validation phase: 861 control subjects, 81 AD patients, 70 vascular dementia patients and 13 frontotemporal dementia patients.	Blood Thiamine diphosphate (TDP), thiamine monophosphate, and thiamine levels	Differential diagnosis	TDP exhibited significant and consistent values for AD diagnosis in both exploration and validation phases. TDP can effectively distinguish AD from vascular dementia.
Bednarska-Makaruk M et al., 2017 [33]	205 patients with dementia (89 with AD, 47 with VaD, 69 with mixed dementia (MD)), 113 persons with MCI and 107 controls.	Serum adiponectin, leptin and resistin levels, IL-6, CRP, chitotriosidase, 25-OH vitamin D, HDL-cholesterol and paraoxonase 1, glucose, insulin and HOMA-IR.	Differential diagnosis	Vascular and mixed dementia are characterized by an increase of resistin. Positive correlation of resistin with inflammation indicators may suggest the potential pro-inflammatory role of resistin in the development of dementia, especially in dementia of vascular mechanism.
Busse M et al., 2017 [34]	60 AD patients, 20 VaD patients, 12 frontotemporal dementia patients and 24 cognitively healthy persons.	Innate and adaptive cell populations in whole blood.	Differential diagnosis	Monocytes and NK cells were diminished in VaD, but not in AD and FTD. B cell and T cell numbers were decreased in all investigated forms of dementia. Changes in the contribution of naïve/memory T cells were only present in AD.
Holm H et al., 2017 [35]	120 AD patients, 83 VaD, 102 MD and 68 other aetiology.	Plasma N-terminal prosomatostatin (NT-proSST)	Risk factor (follow-up period of 4.6 ± 1.3 years)	Higher levels of circulating NTproSST are associated with increased incidence of vascular dementia.
Holm H et al., 2017 [36]	A population-based cohort of 5347 individuals without prevalent dementia	Plasma midregional pro-atrial natriuretic peptide (MR-proANP), C-terminal endothelin-1 (CT-proET-1) and midregional proadrenomedullin (MR-proADM).	Risk factor (follow-up period of 4.6 ± 1.3 years)	Elevated plasma concentration of MR-proANP is an independent predictor of all-cause and vascular dementia. Pronounced increase in CT-proET-1 indicates higher risk of vascular dementia.
Hsu PF et al., 2017 [37]	1436 individuals from a national representative sample in Taiwan.	CRP levels were determined.	Risk Factor (11.04 years (median) of follow-up)	260 individuals (18.11%) were diagnosed with dementia. Those with high CRP had a 55% higher risk of dementia compared with those with normal CRP. After adjusting for possible confounding cardiovascular risk factors, high CRP was independently associated with VaD, but not AD.
Moretti R et al., 2017 [38]	543 patients: 456 patients suffering from subcortical vascular dementia (sVAD), 87 AD and healthy age-matched controls	Clinical laboratory measurements, including serum total cholesterol, triglycerides, and high-density lipoprotein (HDL) cholesterol, have been determined enzymatically and low-density lipoprotein (LDL) cholesterol was calculated using Friedewald’s formula. Serum levels of 25(OH)D, the level of calcium and PTH were measured.The level of folate, vitamin B12 levels and Homocysteine were also tested.	Correlation	Vitamin D deficiency was present in demented cases, as well as low levels of folate and high levels of homocysteine, more pronounced in sVAD cases.
Prabhakar P et al., 2017 [39]	204 patients with small vessel VaD; 200 cognitively normal subjects.	Plasma miRNA profiling.	Differential diagnosis	plasma miR-409-3p, miR-502-3p, miR-486-5p and miR-451a could be used to differentiate small vessel VaD patients from healthy controls.
Quinlan P et al., 2017 [40]	342 patients with subjective or objective mild cognitive impairment	Serum IGF-I concentrations at baseline	Risk factor (mean follow-up: 3.6 years)	In a memory clinic population, low serum IGF-I was a risk marker for subsequent VaD whereas low IGF-I did not associate with the risk of AD. High serum IGF-I was not related to the risk of conversion to dementia.
Suridjan I et al., 2017 [41]	29 possible vascular mild cognitive impairment patients and 89 controls)	Serum lipid peroxidation markers levels. Ratios of early- (lipid hydroperoxides, LPH) to late-stage (8-isoprostane, 8-ISO; 4-hydroxy-2-nonenal, 4-HNE) lipid peroxidation products were calculated.	Correlation with cognitive status	A global effect of group on lipid peroxidation markers was observed, adjusting for sex, years of education, and cardiopulmonary fitness. Lower lipid peroxidation at baseline, as determined by lower 8-ISO concentration, was associated with greater improvement in verbal memory (*F* (1, 64) = 4.738, *p* = 0.03) and executive function (*F*(1, 64) = 5.219, *p* = 0.026) performance. Higher ratios of 8-ISO/LPH and 8-ISO+4-HNE to LPH, were associated with less improvement in executive function performance over a 24-week exercise intervention.
Tang SC et al., 2017 [42]	172 Ischemic Stroke (IS) patients, including 73 with CDR = 0, 63 with CDR = 0.5, and 36 with CDR ≥ 1 (VaD patients).	Plasma concentration of receptor for advanced glycation end products (soluble RAGE (sRAGE); endogenous soluble form of RAGE (esRAGE))	Correlation with cognitive status	Plasma sRAGE and esRAGE were elevated in patients with dementia compared with those without dementia among IS patients.
Vishnu VY et al., 2017 [43]	118 subects, 68 with dementia (MCI-AD and AD: 52; MCI VaSC and VaD:16).	Plasma IL-6; C-Reactive Protein (CRP); plasma fibrinogen; plasma D-dimer.	Differential diagnosis	Plasma Fibrinogen and D dimer: were higher in Vascular group; no difference in IL-6 and CRP.
Wang R et al.,2017 [44]	88 patients with dementia (43 AD patients, 45 VaD patients) and 45 healthy age-matched controls	Plasma Cystatin C (Cys C) and HDL levels	Differential diagnosis	Plasma Cys C levels were higher in patients with AD/VaD than in healthy subjects. Plasma HDL levels were lower in patients with AD/VaD than in healthy subjects.
Yang R et al., 2017 [45]	51 healthy subjects and 41 elderly patients (with 6 participants affected by vascular mild cognitive impairment (Va-MCI)), 9 with VaD, 8 with MCI due to AD and 18 with AD	Plasma GDF-11 and β2-MG levels	Differential diagnosis	No differences in circulating GDF-11 levels amongst the healthy advanced age and four cognitive impairment groups. β2-MG levels increased with age, but there was no significant difference between healthy elderly males and advanced age males. Increased levels of β2-MG were observed in the dementia group compared with the healthy advanced age group.
Brombo G et al., 2018 [46]	320 elderly individuals (≥65 years old): 60 patients with normal cognition; 60 patients with VaD; 100 patients with AD; 100 patients with MCI.	Plasma Klotho levels	Differential diagnosis	Lower levels of plasma Klotho (1st tertile) were associated with higher prevalence of VaD, but not AD.
Latourte A et al., 2018 [47]	1598 people.	Serum Uric Acid (SUA)	Risk Factor (median folow-up duration: 10.1 years)	Association for development of dementia was stronger with vascular or mixed dementia (HR = 3.66 (95% CI 1.29 to 10.41), *p* = 0.015) than Alzheimer’s disease (HR = 1.55 (95% CI 0.92 to 2.61), *p* = 0.10).
Lauriola M et al., 2018 [48]	116 patients: 32 healthy controls, 39 with diagnosis of VaD, 14 MCI and 31 AD.	Erythrocyte associated Aβ (iAβ40 and iAβ42) levels	Differential diagnosis	AD showed different iAβ42 levels as compared to VaD. Conversely, no differences were found for iAβ40.
Shang J et al., 2018 [49]	266 patients with AD, 44 MCI, 33 VaD and 200 ischemic stroke (IS) in comparison to 130 healthy controls.	Plasma fatty acids [eicosapentaenoic acid (EPA) and docosahexaenoic acid (DHA)], adiponectin, reptin, plasma markers of inflammation [high-sensitivity C-reactive protein (hsCRP) and serum amyloid A (serum AA)], and plasma lipids [high-density lipoprotein and low-density lipoprotein (LDL)]	Differential disgnosis	Lower EPA and DHA levels and higher reptin and LDL levels were associated with AD and IS. The reptin/adiponectin ratio was strongly associated with IS. The hsCRP level was more strongly associated with VaD and IS, and the serum AA level was associated with all three cognitive diseases and IS.
Staszewski J et al., 2018 [50]	123 patients (age, mean ± SD: 72.2 ± 8 years, 49% females), with lacunar stroke (*n* = 49), vascular dementia (*n* = 48), and vascular parkinsonism (*n* = 26).	Soluble intercellular cell adhesion molecule-1 (sICAM-1), soluble platelet selectin (sP-selectin), CD40 ligand (sCD40 L), platelet factor-4 (PF-4) and homocysteine; combined high-sensitivity C-reactive protein (hsCRP), interleukin-1α and -6 (IL-1α and IL-6, respectively) and tumor necrosis factor-α (TNF-α).	Correlation with radiological status	Lacunes are associated with different inflammatory markers.
Yang TT et al., 2018 [51]	101 MCI, 107 AD, 30 Parkinson’s disease with dementia (PDD), 20 VaD patients	Serum levels of exosomal miR-135a, -193b, and -384	Differential diagnosis	Both serum exosome miR-135a and miR-384 were up-regulated while miR-193b was down-regulated in AD patients compared with normal controls and non-AD dementias.
Staszewski et al., 2019 [52]	130 patients with marked MRI features of SVD and recent lacunar stroke (*n* = 52,LS), vascular Parkinsonism (*n* = 28,VaP) or dementia (*n* = 50,VaD).	IL-1α, IL-6, hs-CRP, sICAM-1, sP-selectin, TNF-α, homocysteine, fibrinogen, D-dimer, serum total cholesterol (TC), high density lipoprotein cholesterol (HDL-C), low-density lipoprotein cholesterol (LDL-C), triglycerides (TG), eGFR, serum FG, HbA1c, albumin and uric acid (UA).	Risk Factor (mean follow-up time: 22.3 ± 4.3 months)	IL-1α, IL-6, homocysteine, d-dimer were significantly associated with the event of death or stroke, even after adjusting for age, sex and SVD radiological markers.

**Table 2:** CSF biomarkers and VCID.

Author, Year [Reference]	Population	Biomakers	Outcome	Results
Busse S et al., 2014 [53]	serum and CSF of 24 patients with AD, 20 patients with subcortical ischemic vascular dementia (SIVD) and 274 healthy volunteers	N-methyl-D-aspartate glutamate receptors (NMDA-R) autoantibodies directed against the NR1a subunit (NR1a NMDA-R autoantibodies)	Differential diagnosis	The overall seroprevalence was not statistically different between dementia patients and matched controls. CSF samples were negative for NMDA-R autoantibodies.
Herbert MK et al., 2014 [54]	39 DLB, 110 AD, 24 VaD and 28 FTD patients.	CSF concentration of amyloid-b42 (Ab42), total tau protein (t-tau), and phosphorylated tau protein (p-tau) and 3-methoxy-4- hydroxyphenylethyleneglycol (MHPG)	Differential diagnosis	The used combination of Ab42, t-tau, and p-tau could not discriminate among DLB, VaD and FTD. The addition of MHPG to Ab42, t-tau, and p-tau improves the discrimination of DLB from AD, but could not distinguish DLB from other forms of dementia.
Hermann P et al., 2014 [55]	32 patients with VaD with Cerebral Small Vessels Disease (CSVD); 27 patients with AD; 27 patients with AD + CSVD on MRI.	CSF albumin ratio	Differential diagnosis	VaD + CSVD and AD + CSVD had a higher albumin ratio, as an expression of BBB disruption.
Skillbäck T et al., 2014 [56]	107 healthy controls, 223 early onset AD, 1194 late onset AD, 437 subjects with dementia with no other specification,146 FTD, 114 DLB, 517 MD, 45 PDD, 465 VaD, 108 Other)	CSF neurofilament light (NFL) levels	Differential diagnosis	CSF NFL differed among clinical diagnoses, with the highest levels seen in frontotemporal dementia, VaD, and mixed dementia
Ewers M et al., 2015 [57]	55 healthy controls (HC) subjects, 167 patients with AD dementia, 172 subjects with MCI, 22 subjects with subjective memory impairment (SMI), 69 patients with VaD, 26 patients with Lewy body dementia (LBD), 39 patients with FTD, 39 patients with depression, and 86 patients with other neurological disorders (OND).	CSF concentrations of Ab1-42, p-tau181, and total tau.	Differential diagnosis	CSF Aβ1-42 showed the best diagnostic accuracy among the CSF biomarkers. CSF Aβ1-42 discriminates AD dementia from FTD but shows significant overlap with other non-AD forms of dementia, possibly reflecting the underlying mixed pathologies.
Liguori C et al., 2015 [58]	Patients with AD (*N* = 145), healthy controls (*N* = 80) and patients with VaD (*N* = 44).	CSF lactate concentrations, AD biomarker levels (τ-proteins and β-amyloid)	Differential diagnosis	AD patients showed a significant increase of CSF lactate concentration compared to controls and patients with VaD.
Rosenberg GA et al., 2015 [59]	62 patients with Vascular Cognitive Impairment (VCI)	CSF measurements of albumin ratio, matrix metalloproteinases (MMPs), amyloid-β1-42 and phosphorylated-τ181. Patients were followed for an average of 2 years.	Predictor	Inflammatory biomarkers of increased BBB permeability, elevated albumin index and reduced MMP-2 index, predicted the diagnosis of the Binswanger disease (BD) type of subcortical ischaemic vascular disease.
Skillbäck T et al., 2015 [60]	383 Early onset AD and 221 late onset AD patients, 759 vascular dementia, 982 mixed dementia, 232 frontotemporal dementia, 150 Parkinson’s disease dementia and 79 dementia with Lewy bodies (*N* = 79).	cerebrospinal fluid amyloid-β1-42, total tau and phosphorylated tau.	Differential diagnosis	In Parkinson’s disease dementia and vascular dementia low CSF amyloid-β1-42 was associated with low Mini-Mental State Examination score.
Struyfs H et al., 2015 [61]	Patients with AD (*n* = 50), MCI due to AD (*n* = 50) and patients with non-AD dementias (*n* = 50). The non-AD group consisted of 17 patients with FTD, 17 DLB patients, and 16 patients with vascular dementia (VaD). The Control group was composed of 35 subjects.	CSF levels of Aβ isoforms, Aβ(1-37), Aβ(1-38), and Aβ(1-40), as compared to the AD CSF biomarkers Aβ(1-42), T-tau, and P-tau(181P).	Differential diagnosis	Best biomarkers to distinguish AD and VaD were Aβ1-42/T-tau and Aβ 1-42/P-tau181P
Skillbäck T et al., 2017 [62]	Patients diagnosed with Alzheimer’s disease (AD, early onset [EAD, *n* = 130], late onset AD [LAD, *n* = 666]), vascular dementia (VaD, *n* = 255), mixed AD and VaD (MD, *n* = 362), Lewy body dementia (DLB, *n* = 50), frontotemporal dementia (FTD, *n* = 56), Parkinson’s disease dementia (PDD, *n* = 23), other dementias (other, *n* = 48), and dementia not otherwise specified (NOS, *n* = 271), two healthy control groups (n = 292, *n* = 20).	CSF/serum albumin ratio	Differential diagnnosis	Patients with DLB, LAD, VaD, MD, other, and NOS groups had higher CSF/serum albumin ratio than controls. CSF/serum albumin ratio correlated with CSF neurofilament light in LAD, MIX, VaD, and other groups but not with AD biomarkers.
Kiđemet-Piskač S et al., 2018 [63]	152 patients with AD, 28 VaD, and 18 healthy controls (HC).	CSF levels of total tau protein (t-tau), tau protein phosphorylated at threonine 231 (p-tau231).	Differential diagnosis	Total tau levels were significantly elevated in subjects with AD compared to HC, as well as in VaD subjects compared to HC. p-tau_231_ levels were significantly higher in patients with AD vs. HC as well in patients with VaD vs. HC. p-tau231 levels did not distinguish AD from VaD patients.
Barry Erhardt E et al., 2018 [64]	62 possible VCID patients	Matrix metalloproteinases-2 (MMP-2) and MMP-9 in the CSF and plasma and MMP-2 and MMP-9 indexes were calculated. Amyloid b1-42 (Ab42) and phosphoTau181 (PTau) in the CSF.	Diagnostic accuracy	MMP-2 was accurate in predicting VCID diagnosis
Chakraborty A et al., 2018 [65]	age-matched groups of controls with subjective cognitive decline (*n* = 21), AD without the presence of microbleeds (MB) (*n* = 25), AD with MB (*n* = 25), and VaD (*n* = 21) patients.	VEGF levels in CSF	Differential diagnosis	No significant differences were detected between groups

## Abbreviations

VCID	vascular cognitive impairment and dementia
AD	Alzheimer’s disease
CVD	cerebrovascular disease
VaD	vascular dementia
VASCOG	Society for Vascular Behavioral and Cognitive Disorders
BBB	blood–brain barrier
NVU	neurovascular unit
MID	multi-infarct dementia
SVD	small vessels disease
WMHs	white matter hyperintensities
CSF	cerebrospinal fluid
miRNA	MicroRNA
CBF	cerebral blood flow
CAA	cerebral amyloid angiopathy
WMLs	white matter lesions
EPVS	Enlarged Perivascular spaces
MRI	Magnetic Resonance Imaging
ICAM 1	intercellular adhesion molecule-1
VCAM-1	vascular cell adhesion molecule-1
MMP	matrix metalloproteinase
CNS	central nervous system
IL	interleukin
TNFα	Tumor necrosis factor α
TLR4	toll-like receptor 4
CRP	C-reactive protein
ACT	alpha 1-antichymotrypsin
NSE	neuron specific enolase
NMDAR	N-methyl-D-aspartate receptor
MBP	myelin basic protein
NfL	Neurofilament light chain
CADASIL	Cerebral Autosomal Dominant Arteriopathy with Subcortical Infarcts and Leukoencephalopathy
Lp PLA2	lipoprotein-associated phospholipase A2
